# Spontaneous cervical epidural hematoma presenting as quadriparesis: A case report in compliance with the SCARE guidelines

**DOI:** 10.1016/j.amsu.2022.104133

**Published:** 2022-07-14

**Authors:** Salem Al-Dwairy, Alaa Al-Mousa, Jehad Fataftah

**Affiliations:** a Department of Special Surgery, Faculty of Medicine, The Hashemite University, P.O.Box 330127, Zarqa, 13133, Jordan; b Department of Surgery, Prince Hamzah Hospital, Prince Hamzah Street, Al-Rewaq, Amman, 11732, Jordan; c Department of Internal Medicine and Family Medicine, The Hashemite University, P..Box 330127, Zarqa, 13133, Jordan; d Department of Radiology, Prince Hamzah Hospital, Prince Hamzah Street, Al-Rewaq, Amman, 11732, Jordan

**Keywords:** Case report, Cervical epidural hematoma, Quadriparesis, Laminectomy

## Abstract

Spinal epidural hematoma (SEH) is a rare disease. Several pathologies have been described as a cause, including trauma, arteriovenous malformations, coagulopathies, and iatrogenic causes. Spontaneous spinal epidural hematomas (SSEH) are blood in the spinal extradural space without a known cause. The incidence of SSEH has been estimated as 0.1 per 100,000 per year. Herein, we report a case of spontaneous spinal epidural hematoma in the cervical spine.

We report a 57-year-old male patient who presented with sudden axial neck pain associated with upper and lower extremities weakness. Symptoms were precipitated by coughing. MRI of the cervical spine revealed an extradural lesion compressing the dorsal aspect of the spinal cord from C4 – C7. He underwent urgent decompressive laminectomy and evacuation of the hematoma.

## Highlights

• We report a rare case of spontaneous cervical extradural hematoma.
• Patient presented with sudden neck pain and quadriparesis.
• MRI findings were not easy to interpret and to give a final diagnosis.
• Other causes were excluded.
• Urgent decompression was done. Patient improved postoperatively.

## 1 Introduction and importance

Spontaneous spinal epidural hematoma (SSEH) is a rare condition with an estimated incidence of 0.1 per 100,000 individuals [1]. The rarity and difficulty in differentiating it from inflammatory or neoplastic lesions make it an ideal example to report [2].

Spinal epidural hematoma (SEH) results from an accumulation of blood in the spinal epidural space [3] that may compress the spinal cord and nerve roots leading to neurological deficit [4]. It requires early diagnosis and urgent management to achieve recovery of neurologic function [5].

‘Spontaneous’ refers to a non-traumatic etiology and excludes other possible causes such as hemophilia, neoplasms, arteriovenous malformation, coagulopathies, and iatrogenic causes [6,7]. Patients with SEH usually present with acute severe back pain and rapidly progressive signs of the spinal cord or cauda equina compression [8]. MRI is considered to be the modality of choice for diagnosis [7]. In the first 24 hours, SEH usually appears isointense to the cord on T1-weighted images and hyperintense and heterogeneous on T2-weighted images [9]. This makes it difficult to differentiate from inflammatory or neoplastic lesions [2]. By 48 hours, the hematoma intensity will become hyperintense on both T1WI and T2WI [9]. Radiological differential diagnoses include epidural abscess and spinal epidural lymphoma [10]. Patients with residual neurological functions are more likely to show complete recovery [11].

In compliance with the SCARE guidelines [12], we report a case of acute SSEH of the cervical spine, with a past medical history of hypertension and taking 2-Acetoxybenzoic acid for ischemic heart disease. The patient was successfully treated surgically.

## 2 Case presentation

A 57-year-old man presented with acute onset neck pain with rapidly progressive quadriparesis of 9 h duration. Symptoms were precipitated by solid coughing. His past medical history includes hypertension and a drug history of Aspirin.

Neurological examination disclosed quadriparesis. There was no urinary incontinence, and the cranial nerves and cerebellar functions were normal. His vital signs were normal. Blood workup, including platelet count and coagulation profile were normal.

**Fig. 1:**
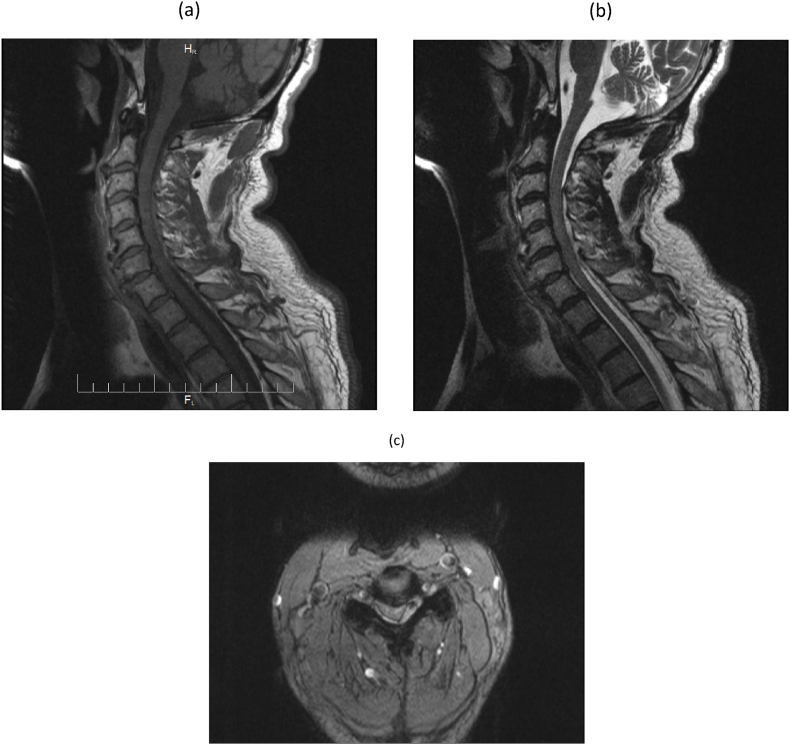
(a) Sagittal T1-weighted image of the cervical spine shows a fluid collection (arrow) in the posterior spinal canal with slight hyperintensity compared with the spinal cord. There is mass effect on the cervical dura mater and spinal cord with anterior displacement. **(b)** Sagittal T2-weighted image of the cervical spine shows a fluid collection (arrow) in the posterior spinal canal with isointense fluid collection along the posterior spinal canal, with displacement of the dura by the posterior epidural collection. **(c)** Axial T2-weighted image at the level of C4C5 shows the posterior fluid collection (white arrow), which appears slightly isointense to the spinal cord with circumferential hypointense hemosiderin deposits (black arrow), and results in external mass effect on the dura mater and displacement of the spinal cord.

An emergent MRI of the cervical spine was performed. The MRI (Fig. 1) showed posterior fluid collection, which appears slightly isointense to the spinal cord with circumferential hypointense hemosiderin deposits, resulting in an external mass effect on the dura mater and displacement of the spinal cord. The final radiological diagnosis was an acute cervical epidural hematoma. Blood workup, including coagulation profile, was normal.

Provisional differential diagnosis was discussed with the patient. Risks and benefits were addressed, and the prognosis was clarified.

The patient underwent an emergency operation done by a senior neurosurgeon. We did a C4 to C7 decompressive laminectomy and hematoma evacuation. Grossly, a well-organized clot was found in the corresponding levels, extending posterio-laterally. No other pathologies were detected intraoperatively, such as vascular malformation. No obvious bleeding source was identified, and hemostasis was achieved easily.

The histopathology analysis of the specimen was suggestive of a hematoma, with no evidence of abnormal blood vessels suggesting the presence of any vascular malformation.

The patient was diagnosed with a spontaneous epidural hematoma, after exclusion of the presence of coagulopathy, and vascular malformation on gross histopathology, and with no history of trauma.

Postoperatively the motor power in all the limbs improved within 24 hours. The patient was discharged four days after surgery without any complications. He was transferred to a rehabilitation unit for physical therapies.

## 3 Clinical discussion

SSEH is a rare neurosurgical pathology with an estimated incidence of 0.1 per 100,000 individuals [1]. They are considered surgical emergencies [13]. It can occur spontaneously or after trauma or iatrogenic procedures. Other causes include coagulopathy, vascular malformations, tumors, and cavernous angiomas [1,10,11]. Presentation depends on the location of the hematoma and the degree of spinal cord compression. Most patients present with sudden severe neck or back pain, often with radicular symptoms, followed by motor or sensory deficits that might progress depending on the rapidity and severity of the bleeding [10,14].

The source of bleeding is considered venous in origin due to the absence of sphincters in the spinal epidural veins, thus making them prone to bleed after pressure changes. This is correct when talking about thoracic or lumbar hematomas. Due to low venous pressure in the cervical spine, this theory is thought to be invalid. A second theory owes the bleeding from free epidural anastomotic arteries in connection with radicular arteries in the cervical spine [13,15].

MRI is the modality of choice for diagnosis. In the first 24 hours, hematoma usually appears isointense to the cord on T1WI and hyperintense and heterogenous on T2WI. Then hematoma usually becomes hyperintense on T1WI and T2WI during the next 48 hours. Chronic hematomas appear hypointense on both T1WI and T2WI [9,16]. [17]. Radiological differential diagnoses include epidural abscess and spinal epidural lymphoma [10].

Urgent surgical intervention is warranted within 12–48 hours of symptoms onset [[18], [19], [20]]. Outcomes typically are poor without surgical intervention [21]. Prognosis mainly relies on the preoperative neurological status, where patients with residual neurological function at presentation carry a better prognosis [10,13]. Non-surgical management is reserved only for asymptomatic patients, where strict bed rest and serial imaging studies are recommended [19].

## 4 Conclusion

Spontaneous spinal epidural hematoma (SSEH) is a rare condition. They are considered a surgical emergency. MRI is the modality of choice for diagnosis. Surgical intervention is the ideal method for achieving complete recovery. Non-surgical management is reserved only for asymptomatic patients. As in our case, spinal cord compression due to hematoma after urgent surgical intervention can benefit from rehabilitation. This may lead to significant improvement in neurological functions.

## Provence and peer review

Not commissioned, externally peer-reviewed.

## Ethical approval

This study was approved by the Ethical Committee of Prince Hamzah hospital.

## Sources of funding

This work received no specific grant from any funding agency in the public, commercial, or not-for-profit sectors.

## Author contributions

Salem Al-Dwairy: Diagnosed and treated the case, wrote the largest share of the repot.

Ala l-Mousa: writing the paper and critical revision of the work.

Jehad Fataftah: final approval of the version be published.

## Registration of research studies

1. Name of the registry:
2. Unique identifying number or registration ID:
3. Hyperlink to your specific registration (must be publicly accessible and will be checked):

## Guarantor

The author accepts full responsibility for this work, had access to the data and controlled the decision to publish.

## Consent

A written informed consent was obtained from the patient for publication of this case report and accompanying images.

## Declaration of competing interest

All authors have no conflicts of interest to declare.
